# Supplementary material for: Sensitive near point-of-care detection of asymptomatic and submicroscopic Plasmodium falciparum infections in African endemic countries
Source: Nat Commun. 2025 Oct 10;16:8925. doi: 10.1038/s41467-025-64027-4 (PMC12514294; doi:10.1038/s41467-025-64027-4)

Sensitive Near Point-of-Care Detection of Asymptomatic and Sub-microscopic *Plasmodium falciparum* Infections in African Endemic Countries

Dimbintsoa Rakotomalala Robinson[1,&], Ivana Pennisi[2,3,&], Matthew L. Cavuto[2,3,&], Francois Kiemde[4], Martin Chamai[5], Diane Yrgnur Some[4], Elliot Quigley[3], Kenny Malpartida-Cardenas[2,3], Mamadou Ousmane Ndiath[1], Simon Correa[1], Bubacarr Darboe[1], Lindsay B. Stewart[6], Pantelis Georgiou[7], Mamadu Baldeh[1], Halidou Tinto[4], Aubrey J. Cunnington[2,8], Annette Erhart[1], Umberto D'Alessandro[1], Jesus Rodriguez-Manzano[2,3,9,§], on behalf of the NIHR Global Health Research Group on Digital Diagnostics for African Health Systems*

[1] MRC Unit The Gambia at the London School of Hygiene and Tropical Medicine, Banjul, The Gambia
[2] Department of Infectious Disease, Faculty of Medicine, Imperial College London, London, UK
[3] ProtonDx, Translation & Innovation Hub, Imperial College London, London, UK
[4] Unité de Recherche Clinique de Nanoro, Institut de Recherche en Sciences de la Santé, Nanoro, Burkina Faso
[5] West African Centre for Cell Biology of Infectious Pathogens, College of Basic and Applied Sciences, University of Ghana, Legon, Accra, Ghana
[6] Faculty of Infectious and Tropical Diseases, London School of Hygiene & Tropical Medicine, London, UK
[7] Department of Electrical and Electronic Engineering, Imperial College London, London, UK
[8] Centre for Paediatrics and Child Health, Imperial College, London, UK
[9] The Fleming Initiative, Imperial College London and Imperial College Healthcare NHS Trust, London, UK
[&] These authors contributed equally
[§] Corresponding author: j.rodriguez-manzano@imperial.ac.uk
* A list of authors appears at the end of the paper

Table of Contents

Supplementary Table 1. Primer sequences of the LAMP assays used in this study 2

Supplementary Table 2. Evaluating the Dragonfly system specificity at various reaction time points 3

Supplementary Figure 1. Confusion matrices for asymptomatic samples 4

Supplementary Table 3. Comparison of the Dragonfly Pan/*Pf* malaria platform with malERA Target Product Profiles (TPP) Criteria for Community-Level Malaria Screening 5

Supplementary Table 4. Comparison of the characteristics of the Dragonfly platform and the two commercial Malaria LAMP technologies 7

Supplementary Table 5. Preliminary cost analysis of prototype approach, including both consumable and labour costs per sample, compared against market pricing for Alethia® Malaria Tests and required incubation and detection instrument 8

Supplementary Figure 2. Giemsa-stained thin blood film of the final culture preparation, showing red blood cells (RBCs) singly infected with *Plasmodium falciparum* parasites at the ring stage 9

Supplementary Table 6. Equations used to calculate sensitivity and specificity for values reported in the manuscript 10

Supplementary Methods 11

Supplementary Table 1. Primer sequences of the LAMP assays used in this study.

Target	LAMP assay	Oligonucleotide sequence (5' to 3')
Pan/*Pf*	LAMP-PfK13_F3	GGAGCAGCTTTTAATTACCTT
	LAMP-PfK13_B3	ATGACATGAATTTAGAACTTCGCC
	LAMP-PfK13_LF	AATATGTTATGTTCATTATCAA
	LAMP-PfK13_LB	GAGAAAAAAATGAATTTTGGAGCT
	LAMP-PfK13_FIP	TGGTTGATATTGTTCAACGGAATCT-ATCAAATATATGTTGTTGGAGGT
	LAMP-PfK13_BIP	TGGCAATTTCTA AATGGTGTACCA-ATAAGAATCTGACAATGTGGC
	Polley-Pf_F3	CTCCATGTCGTCTATCGC
	Polley-Pf_B3	AACATTTTTTAGTCCCATGCTAA
	Polley-Pf_LF	CGGTGTGTACAAGGCAACAA
	Polley-Pf_LB	GTTGAGATGGAAACAGCCGG
	Polley-Pf_FIP	ACCCAGTATATTGATATTGCGTGAC-AGCCTTGCAATAAATAATATCTAGC
	Polley-Pf_BIP	AACTCCAGGCGTTAACCTGT-AATGATCTTTACGTTAAGGGC
	LAMP-Pan18s_F3	GTATCAATCGAGTTTCTGACC
	LAMP-Pan18s_B3	CTTGTCACTACCTCTCTTCT
	LAMP-Pan18s_LF	CGTCATAGCCATGTTAGGCC
	LAMP-Pan18s_LB	AGCTACCACATCTAAGGAAGGCAG
	LAMP-Pan18s_FIP	TCGAACTCTAATTCCCCGTTACC-TATCAGCTTTTGATGTTAGGGT
	LAMP-Pan18s_BIP	CGGAGAGGGAGCCTGAGAAA-TAGAATTGGGTAATTTACGCG

The tandem Pan/*Pf* assay targets both Pan-malaria DNA sequences, conserved across multiple *Plasmodium* species responsible for malaria, and *P. falciparum*-specific sequences.

Supplementary Table 2. Evaluating the Dragonfly system Specificity at Various Reaction Time Points.

Reaction Time	True Negatives (TN)	False Positives (FP)	Total Negative Samples	Specificity (%)	95% CI (%)
50 min	118	2	120	98.3	96.0 – 100
55 min	118	2	120	98.3	96.0 – 100
60 min	118	2	120	98.3	96.0 – 100

The analytical specificity of the Dragonfly Test was evaluated at different reaction time points. At 50 minutes, the assay demonstrated a specificity of 98.3% (95% CI: 96.0–100.6%), with 2 false positive results out of 120 negative samples tested. Extending the reaction time up to 60 minutes resulted in the same performance indicating the test maintains high specificity even with extended incubation periods over the maximum reading time.

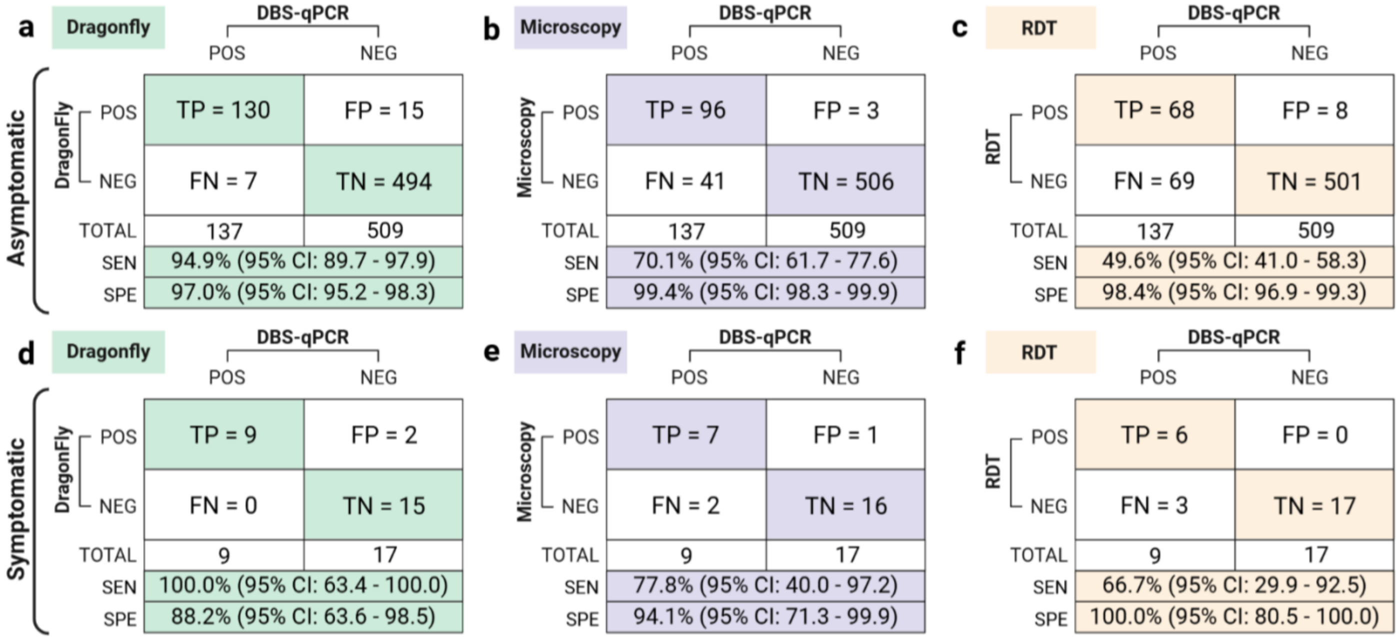

Supplementary Figure 1. Comparison of the clinical performance of Dragonfly, microscopy, and RDT methods using whole blood finger prick samples divided by asymptomatic and symptomatic cases, with DBS-qPCR as the gold-standard comparator. For each group, the number of true positives, total positive cases, sensitivity rate with 95% CI, number of true negatives, total negative cases, and specificity rate with 95% CI are provided. Created in BioRender. Cavuto, M. (2025) https://BioRender.com/rwbh4tn. Source data are provided as a **Source Data file**.

Supplementary Table 3. Comparison of the Dragonfly Pan/*Pf* malaria platform with malERA Target Product Profiles (TPP) Criteria for Community-Level Malaria Screening

MalERA Criteria for Screening/Surveillance (District Level or Below)		Status
**Technical specifications**		
Analytic sensitivity (parasite/μL)	E=20, D≤5	
Diagnostic sensitivity	E>95%, D≥99%	
Analytic specificity	Negative all pathogens, common blood disorders	
Diagnostic specificity	E>99% surveillance low-transmission areas, E>95% screening	
Temperature stability	E, 30˚ C; D, 45˚ C for short periods	
Integrity of packaging	E, Moisture-proof	
**Species detection/differentiation**		
P.f predominant areas	E, P.f; D, P.f/pan	
P.f and non-P.f areas	E, P.f/pan; D, differentiation all species	
Genotyping	No/O	
Ability to detect gametocytes	O	
Ability to detect hypnozoites	D	
**Health systems and technical specifications**		
Packaging of tests or reagents	D, all required consumables enclosed; D, bulk packaging displays temperature violations	
Field stability/shelf life of consumables	E, 12 months (6 months since country); D, 2 y from manufacture (≥18 months in country)	
Training requirements	D, <1 week of pretrained medical technician	
Reagent requirements	E, nontoxic, all nonroutine provided; D, all necessary consumable items to perform the test provided in the kit	
Invasiveness	E, finger prick or less; D, non-invasive	
Rapidity of results	E≤2 days; D≤ half-day	
Ease of use	E, within medical tech ability; D, simple, few steps	
Cost	D≤US$1 per test	
Safety	E, high blood safety with basic universal precautions	
Waste disposal	Basic health system waste disposal	
Inter-reader reliability (clarity of result)	Kappa.0.9	
Instrumentation and laboratory infrastructure requirements	D, all provided with test	
D = desirable, E = essential, O = optional		

Desirable (D) criteria met	Essential (E) criteria met	Criteria not met	Data unavailable or not applicable

Supplementary Table 4. Comparison of the characteristics of the Dragonfly platform and the two commercial Malaria LAMP technologies. (Loopamp™ Malaria and Alethia® Malaria)

Characteristics	Alethia® Malaria	Loopamp™ Malaria	Dragonfly Malaria Pan/*Pf* platform
**General specifications and performances**			
Targets	Pan-Plasmodium	Pan-Plasmodium; *P.falciparum; P.vivax*	Pan-Plasmodium /*P.falciparum*
Analytical sensitivity for *Plasmodium falciparum*	0.7-2 parasites/µL	1–2 parasites/µL	0.6 parasite/µL
Diagnostic sensitivity in symptomatic patients	97.2 % (95CI: 92.6–99.1)[41]	97.0 % (95CI: 89.6–99.6)[42]	100 % (95CI: 63.4-100)
Diagnostic sensitivity in asymptomatic infected individuals	N/A	83.3 % (95CI: 58.6-96.4) [a] [39]<br>72.2 % (95CI: 62.6–80.2) [b][43]<br>40.8 % (95CI: 27.0–55.8) [c][40]	94.9 % (95CI: 89.7-97.9)
Specificity	87.7 % (95CI: 76.6–94.2)[41]	99.7 % (95CI: 99–100) [a] [39]<br>99.9 % (95CI: 99.8–100) [c][40]<br>99.2 % (95CI: 98.1–99.7) [42]	96.8 % (95CI: 94.9-98.0)
Power requirements	Mains power	Mains power or portable battery	Mains power or portable battery
**DNA extraction**			
DNA extraction kit	Alethia Malaria Specimen Preparation orAlethia Malaria PLUS Specimen Preparation	Loopamp™ PURE DNA Extraction Kit (optional) or [a]	SmartLid Blood DNA/RNA Extraction Kit
DNA extraction technology	Filtration-based method	Heating step + adsorbent powder (Loo)pamp™ PURE DNA	Magnetic beads-based extraction
Sample type	Whole blood collected in EDTA	Whole blood or dried blood spots	Whole blood collected in EDTA
Sample volume	50 µl	30 µl	100 µl
Required equipment	None	HumaHeat Incubator [d] or HumaLoop M [e]	PDX Heat block or Regular Heat block
Weight of equipment	NA	HumaHeat :1.8 kg	1.03 kg
Dimensions of equipment	NA	HumaHeat : 15 cm × 17 cm × 14 cm	14 cm × 11 cm × 13 cm
**DNA amplification**			
Required equipment	Alethia® Incubator Reader (Illumipro-10™ incubator/reader previously)	HumaLoop M [e] or HumaTurb A +C [f]	PDX Heat block or Regular Heat block
Weight of equipment	2.95kg	HumaLoop M [e]: 9.5 kg or HumaTurb A+C [f]: 2.5 kg	1.03 kg
Dimensions of equipment	21 cm x 29.2 cm x 9.5 cm	HumaLoop M [e]: 25 cm × 27.6 cm × 18.2 cm<br>HumaTurb A+C [f]: 52 × 35 × 23.5 cm	14 cm × 11 cm × 13 cm
Throughput (max number of samples/run)	10	HumaLoop M: 16<br>HumaTurb A: 16	24
Recommended incubation time	40 minutes	40 minutes	Min 20 minutes, Max 40 minutes
Reagents	Lyophilised	Lyophilised	Lyophilised
**Result readout**			
Detection method	Reaction solution absorbance characteristics	Fluorescence under UV light or turbidity	Colorimetric based on pH change
Required equipment	Alethia® Incubator Reader	HumaLoop M [e] or HumaTurb A+ C [f]	Naked eye

Output	Qualitative	Qualitative	Qualitative

[a] Using boil & spin extraction method[b] Using Chelex as extraction method
[c] Using HTP (High throughput) extraction method[d] The HumaHeat Incubator is required when using HumaTurb A+C for amplification and result readout.
[e] The HumaLoop M integrates both sample preparation, amplification and result readout.
[f] The HumaTurb A+C consists of two units: the HumaTurb A (amplification unit) and the HumaTurb C (control unit).

Comparison of Dragonfly with two Commercial LAMP Technologies. Dragonfly exhibits analytical sensitivity of 0.6 parasites/µL for *P. falciparum*, compared to 1-2 parasites/µL for Loopamp and 0.7-2 parasites/µL for Alethia. In symptomatic patients, Dragonfly demonstrated high diagnostic sensitivity (100%), comparable to Loopamp™ (97.0%) and Alethia® (97.2%)**.** For asymptomatic individuals, sensitivity data are only available for Dragonfly (94.9%) and Alethia (40.8-83.3%). All three platforms target either pan-*Plasmodium* or both pan-*Plasmodium* and *P. falciparum*, and can operate using mains power or portable batteries. Dragonfly requires only two regular heat blocks making it the lightest and most compact option. In contrast, Loopamp and Alethia rely on dedicated incubators and readers, increasing their overall weight, size and cost. While market price per test is not yet available for Dragonfly, preliminary cost analysis estimates the prototype at £4.01 per test. Loopamp appears more cost-effective at approximately €5.2 per test, compared to Alethia at £43.

Supplementary Table 5. Preliminary cost analysis of prototype approach, including both consumable and labour costs per sample, compared against market pricing for Alethia® Malaria Tests and required incubation and detection instrument.

**Component**	**Alethia® Malaria** *(approximate market cost, £)*	**Dragonfly Malaria Pan/*Pf*** *(estimated manufacturing cost, £)*	
Test cost per sample	43[1]	Consumable test components (e.g. reagents, plastics, packaging, etc.)	2.68
		Labour (UK-based, assuming batch sizes of <10K reactions)	1.33
		Total test cost per sample	4.01
Instrumentation cost (e.g. for reaction incubation and result readout)	16,000[2]	Isothermal heat block (for both sample lysis and LAMP incubation)	112.00

[1] https://www.szabo-scandic.com/en/alethia-malaria
[2] https://www.fishersci.com/shop/products/alethia-incubator-reader/23029014

Supplementary Figure 2. In vitro cultured ring stages (3D7 strain).

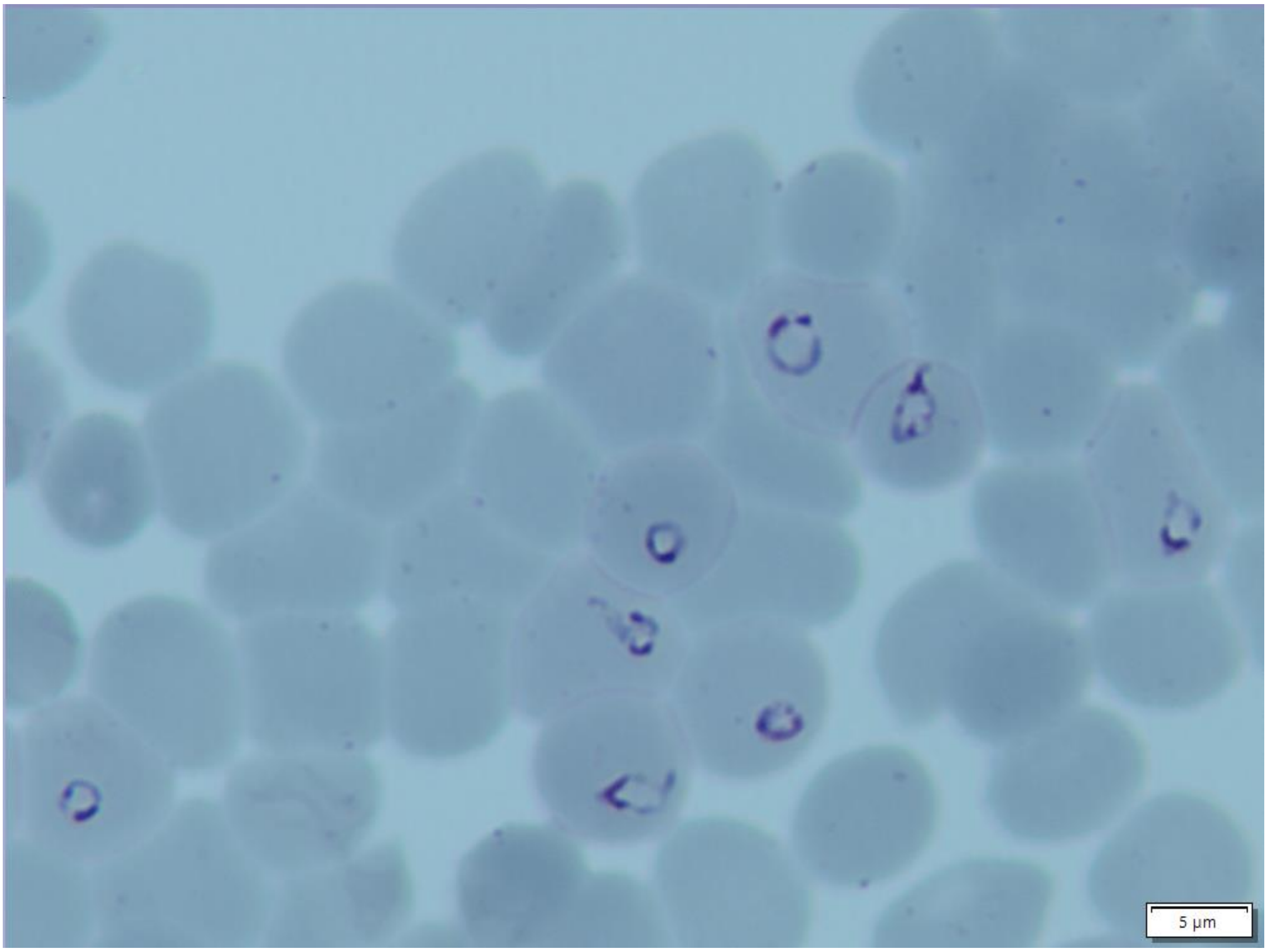

Giemsa-stained thin blood film of the final culture preparation, showing singly infected red blood cells (RBCs) at the ring stage.

Supplementary Table 6. Equations used to calculate sensitivity and specificity for values reported in the manuscript.

	**Comparator**	
**Test**	Positive	Negative
Positive	TP	FP
Negative	FN	TN
Total	$TP + FN$	$FP + TN$
Sensitivity	$\frac{TP}{TP + FN}$	
Specificity	$\frac{TN}{TN + FP}$	

Supplementary Methods

Protocol for high-throughput Extraction of DNA/RNA from Whole Blood with SmartLid and Detection with Dragonfly Malaria Pan/*Pf*

REQUIRED MATERIALS

- ProtonDx SmartLids
- ProtonDx Magnetic Keys
- ProtonDx 20 uL fixed volume Pipettes and tips
- ProtonDx Vortex mixer (optional)
- ProtonDx SmartLid Rack (optional)
- ProtonDx SmartLid Vortex Tool (optional)
- ProtonDx Heat block (locked to 63.5°C)
- Microcentrifuge tubes (2.0 mL)
- ProtonDx Lysis Binding Buffer
- ProtonDx Wash I concentrate
- ProtonDx Wash II concentrate
- ProtonDx Proteinase K
- ProtonDx Magnetic Beads
- ProtonDx Elution buffer
- Dragonfly Malaria Pan/*Pf* panels & result cards
- Molecular biology grade ethanol (>99%)
- Personal protective equipment PPE
- Decontamination solutions
- 1000 uL pipette and sterile filter tips
- Waste collection bag
- Timer
- EDTA collection tubes for whole blood

IMPORTANT Before Starting

- Always wear PPE, including lab coat and gloves. Change gloves if they touch anything not sterile.
- Prior to performing any biological procedure, ensure the working environment is clean using a decontamination spray according to local guidelines.
- Always use pipette tips that have a filter and are sterile.
- **Add 18 mL of molecular biology grade ethanol (>99%) to the Wash I (concentrate) bottle and 21 mL to the Wash II (concentrate) bottle** as indicated on the label.

SETUP for Multiple Extractions

1. Set up the required number of flip-cap tubes in a SmartLid Rack, with different rows for:

   Lysis (Row A)

   Wash I (Row B)

   Wash II (Row C)

   Elution (Row D)

   (For example, 6 extractions will require a total of 24 tubes, split in 4 rows of 6 tubes.)

2. If extracting from a **liquid sample**, including whole blood stored in EDTA tubes, prepare a master mix of Lysis Binding Buffer and Magnetic Beads in a 50 mL falcon tube according to the table below. Multiply the volume per tube by the number of extractions (n) and include an additional 10% to allow for pipetting errors:

Component	Volume per tube [1]	Volume of Master Mix for **n** extractions
Lysis Binding Buffer	600 µL	600 µL × **n**
Magnetic Beads	40 µL	40 µL × **n**
**Total volume**	640 µL	640 µL × **n**

[1] Use 10% overage calculation when making a master mix for use with multiple samples.

3. If needed, Negative extraction control (EDTA whole blood from healthy control) and Positive extraction control (EDTA whole blood spiked with malaria parasites) can be added for each user performing the Dragonfly test.

4. Prefill all tubes according to the table below:

Tube/Step	Components and Volumes
A: Lysis Binding	**640 µL** Lysis Binding Master Mix
B: Wash I	**500 µL** Wash Buffer I [1]
C: Wash II	**500 µL** Wash Buffer II [1]
D: Elution	**50-200 µL** Elution Buffer

[1] Ensure that molecular biology grade ethanol (>99%) has been added to the Wash Buffer (concentrate) as indicated on the bottle.

LYSIS BINDING

1. Add **20 µL** of Proteinase K to each Lysis tube (prefilled with Lysis Binding Buffer and Magnetic Beads master mix).

2. Add **100 µL** of whole blood to the Lysis tube.

3. Close with a SmartLid (without a Magnetic Key inserted) and briefly vortex the tube (**1-2 seconds**) to mix.

4. Incubate the lysis tube (with SmartLid inserted) at **65°C (or 63.5 if using ProtonDx Heat Block)** for **5 minutes**.

5. Remove the SmartLid and add **400 µL** of EtOH (>99%) to the lysed sample.

6. Return the SmartLid (still without the Magnetic Key) to the tube and pulse-vortex for **60 seconds**.

   Note: For processing multiple samples at a time, the SmartLid Vortex Tool (100174) can be used for each mixing step. Ensure the retainer disk is engaged securely before vortexing.

7. Insert a Magnetic Key into the SmartLid, turning it 90 degrees clockwise to lock, and invert the tubes several times to collect all Magnetic Beads onto the SmartLid.

   Allow the tube to remain upside down for **~30-60 seconds** after the first inversion, followed by multiple quicker (**~5-10 seconds each**) inversions to ensure all beads are collected.

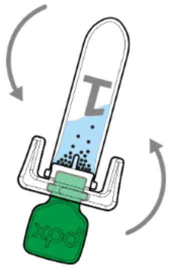

WASH

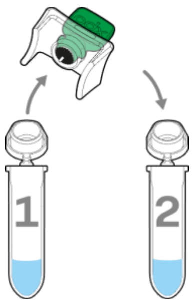

1. Transfer the SmartLid (with Magnetic Key INSERTED) from the lysis tube into the corresponding Wash I tube, ensuring it's firmly inserted in the wash tube.

   Important: Ensure the Magnetic Key is inserted during transfer to avoid losing Magnetic Beads and captured nucleic acids.

2. Remove the Magnetic Key and pulse-vortex for **60 seconds** to resuspend the Magnetic Beads

3. Insert/lock the Magnetic Key into the SmartLid and invert the tube several times to collect all Magnetic Beads onto the SmartLid. **Ensure the liquid is clear before proceeding.**

   Tip: Initially shake the tube with the Magnetic Key inserted to first resuspend all Magnetic Beads, followed by gentle inversions for collection.

4. Repeat steps 1-3 for Wash II, transferring, resuspending, mixing, and again collecting the beads.

5. Once all washing steps are complete, and all Magnetic Beads are collected onto the SmartLid, remove the SmartLid and set it down (Magnetic Beads facing down) on a clean surface for **60 seconds** to **allow all EtOH to evaporate.**

ELUTION

1. Once the evaporation step is complete, insert the SmartLid (with Magnetic Key still INSERTED) into the corresponding Elution tube.

2. Remove the Magnetic Key and mix/shake the tube for **60 seconds** to fully resuspend the beads.

   Important: Do **NOT** use the vortex tool and vortex mixing for the elution step. Shake the tubes instead.

3. Insert/lock the Magnetic Key into the SmartLid. **Ensure the liquid is clear before proceeding.**

4. Flick the tube down to collect as much elution volume as possible, discard the SmartLid and attached Magnetic Beads, and store the elution tube. **Please do not discard the green Magnetic keys, as they can be cleaned and reused.**

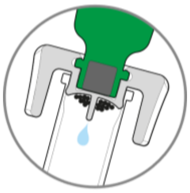

TEST PANEL LOADING

Once the extractions are complete testing should be performed using the DF Malaria test panel (Pan/*Pf*) and dedicated heating block.

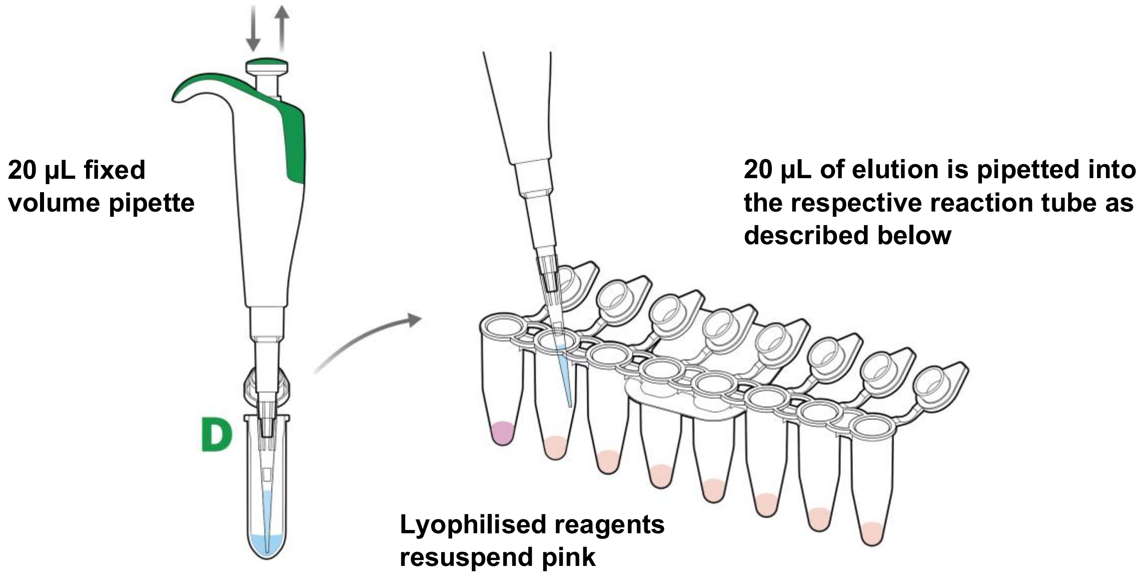

1. With a new pipette tip, rehydrate reaction **tubes 1 and 8** of the Pan/*Pf* test panels with 20 µL of the **Negative extraction control**, using the ProtonDx fixed volume pipette. Discard tip.

2. With a new pipette tip, rehydrate reaction **tubes 2 to 7** with 20 µL of the elution's from **each patient sample**, making sure to **use a new tip for every sample**. Please consider each malaria strip can test up to 6 patients (P1 to P6) plus controls.

3. Flick down the Dragonfly reaction tubes to make sure all the liquid sits at the bottom.

   Important: Close lids securely!

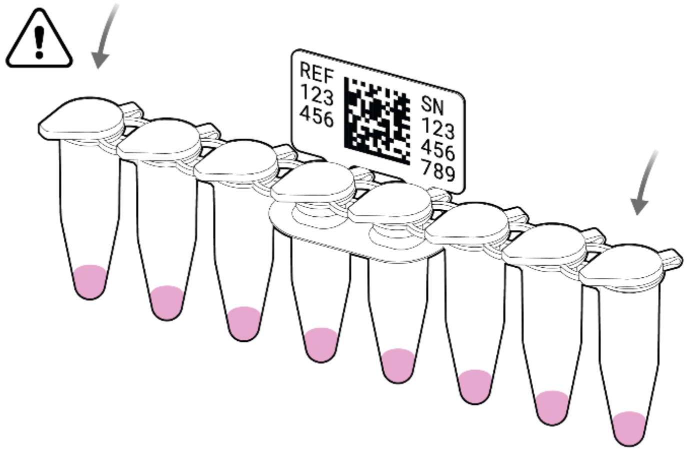

4. Transfer the DragonFly reaction tubes into the pre-heated ProtonDx heat block, after ensuring that the heater has reached 63.5°C making sure all the lids are securely closed.

5. Incubate the strip for 40 minutes.

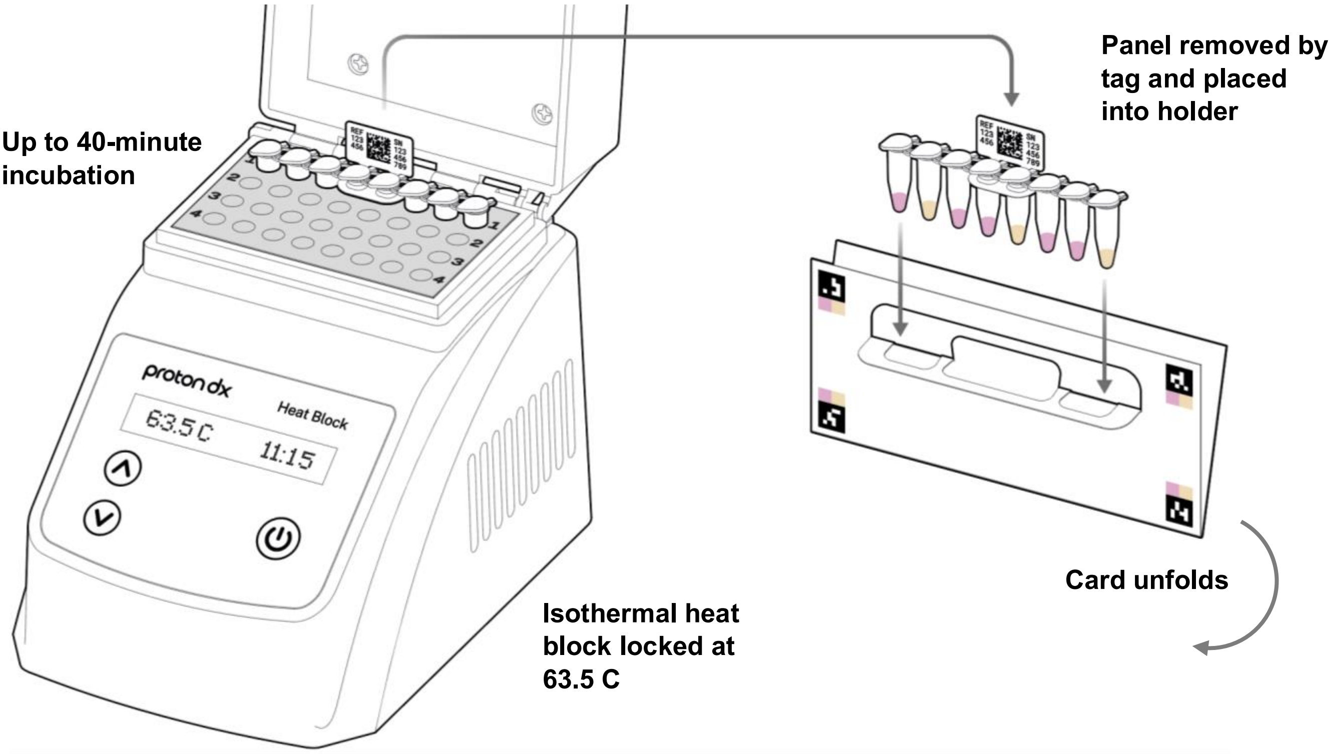

6. After the time has elapsed remove the DragonFly reaction tubes from the heater and place them on the results card.

7. Leave the strips to cool at room temperature for 10 seconds prior to taking a photo of the results.

RESULT INTERPRETATION

The user interpreting the panel should have good colour vision and be under good lighting conditions. A pen may be used to mark your selections and write additional details onto the card. **Write the Sample ID, Date, and operator's name on the card.** Insert the test panel into the card and fold open to show the result matrix.

Important: Always be sure the tube lids remain closed.

The Malaria Pan/*Pf* panel has 2 controls, and targets.

Outcomes will be either Pink or Yellow.

- The **tube 1** is a colour control and **must stay pink**. If it turns yellow, the test is invalid. Load the negative extraction control in tube 1.
- Tube 8 is an internal control and must turn yellow. The test is invalid if it remains pink.
- **Tubes 2, 3, 4, 5, 6, 7** are the targets and will be **either yellow or pink**. Yellow indicates the target is detected, or positive, and pink indicates the target is not detected, or negative.

If the controls all match the expected colours, the test is valid.

Outcomes will be either pink (negative) or yellow (positive)

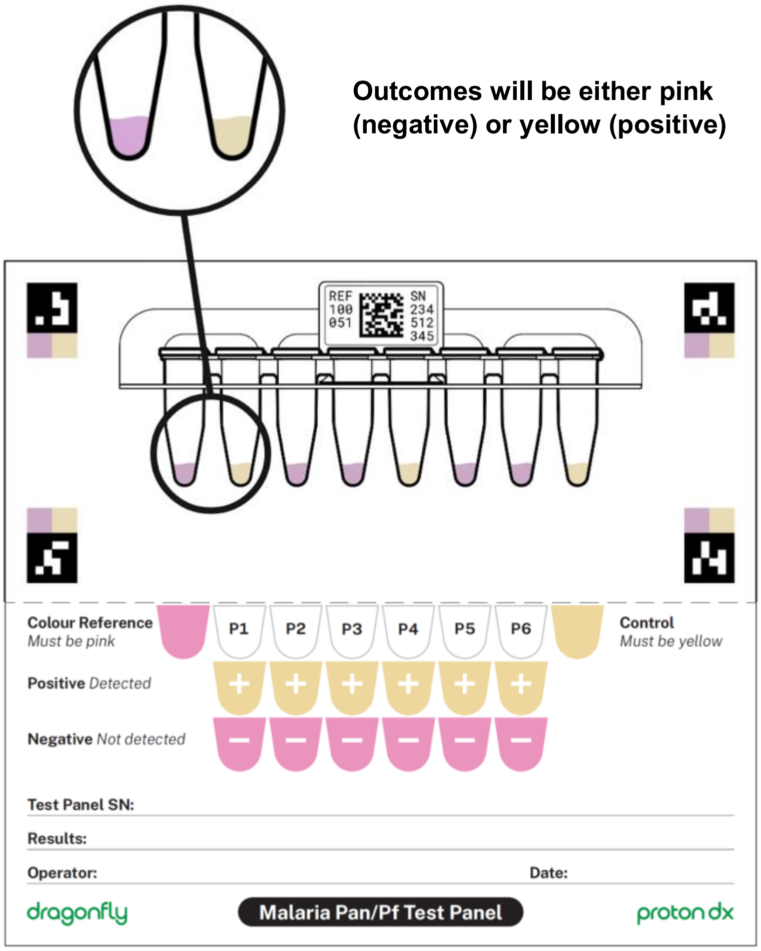

Supplement: Supplementary file 1 — Supplementary Information [file 41467_2025_64027_MOESM1_ESM.pdf]
