# Supplementary material for: Sensitive near point-of-care detection of asymptomatic and submicroscopic Plasmodium falciparum infections in African endemic countries
Source: Nat Commun. 2025 Oct 10;16:8925. doi: 10.1038/s41467-025-64027-4 (PMC12514294; doi:10.1038/s41467-025-64027-4)
Supplement: Supplementary file 2 — Description of Additional Supplementary Information [file 41467_2025_64027_MOESM2_ESM.pdf]

Description of Additional Supplementary Files

File Name: Supplementary Data 1
Description: Raw data from Dragonfly tests performed on *Plasmodium*-negative blood samples. Each row represents a single repetition, with associated data including Dragonfly test result, incubation time (50, 55 and 60 minutes), test control status, test date, and corresponding Picture ID.

File Name: Supplementary Data 2
Description: Raw data used to assess the analytical sensitivity of malaria detection using Alethia®. Each row includes the test ID, parasite density (parasites/µL), corresponding Alethia test result, and the test date.

File Name: Supplementary Data 3
Description: Raw data used to assess the analytical sensitivity of malaria detection using Dragonfly. Each row includes the test ID, the parasite density (parasites/µL), the test control status, corresponding Dragonfly test result, and the test date.

File Name: Supplementary Data 4
Description: Raw data used to assess the analytical sensitivity of malaria detection using DBS-qPCR. Each row includes the test ID, the parasite density (parasites/µL), and the corresponding DBS-qPCR test result.

File Name: Supplementary Data 5
Description: Raw data used to assess the analytical sensitivity of malaria detection using WB-qPCR. Each row includes the test ID, the parasite density (parasites/µL), and the corresponding WB-qPCR test result.

File Name: Supplementary Data 6
Description: Raw data from samples collected from both symptomatic and asymptomatic individuals tested as part of the clinical validation of the presented Dragonfly Pan*/Pf* Malaria method. Each row corresponds to an anonymised sample, with associated data including the survey of origin, sex, age, malaria symptomatic status, RDT brand, RDT result, LM result with parasite density (parasites/µL) and *Plasmodium* species, Dragonfly test control status, Dragonfly panel type, Dragonfly test date, Dragonfly test result, and DBS-qPCR result with corresponding Ct value.
