# Supplementary material for: Sensitive near point-of-care detection of asymptomatic and submicroscopic Plasmodium falciparum infections in African endemic countries
Source: Nat Commun. 2025 Oct 10;16:8925. doi: 10.1038/s41467-025-64027-4 (PMC12514294; doi:10.1038/s41467-025-64027-4)
Supplement: Supplementary file 9 — Reporting Summary [file 41467_2025_64027_MOESM9_ESM.pdf]

nature portfolio

Corresponding author(s): Dr Jesus Rodriguez Manzano

Last updated by author(s): 07/08/2025

Reporting Summary

Nature Portfolio wishes to improve the reproducibility of the work that we publish. This form provides structure for consistency and transparency in reporting. For further information on Nature Portfolio policies, see our Editorial Policies and the Editorial Policy Checklist.

Statistics

For all statistical analyses, confirm that the following items are present in the figure legend, table legend, main text, or Methods section.

n/a	Confirmed	
☐	☒	The exact sample size (*n*) for each experimental group/condition, given as a discrete number and unit of measurement
☐	☒	A statement on whether measurements were taken from distinct samples or whether the same sample was measured repeatedly
☐	☒	The statistical test(s) used AND whether they are one- or two-sided<br>*Only common tests should be described solely by name; describe more complex techniques in the Methods section.*
☒	☐	A description of all covariates tested
☒	☐	A description of any assumptions or corrections, such as tests of normality and adjustment for multiple comparisons
☐	☒	A full description of the statistical parameters including central tendency (e.g. means) or other basic estimates (e.g. regression coefficient) AND variation (e.g. standard deviation) or associated estimates of uncertainty (e.g. confidence intervals)
☐	☒	For null hypothesis testing, the test statistic (e.g. *F*, *t*, *r*) with confidence intervals, effect sizes, degrees of freedom and *P* value noted<br>*Give P values as exact values whenever suitable.*
☒	☐	For Bayesian analysis, information on the choice of priors and Markov chain Monte Carlo settings
☒	☐	For hierarchical and complex designs, identification of the appropriate level for tests and full reporting of outcomes
☒	☐	Estimates of effect sizes (e.g. Cohen's *d*, Pearson's *r*), indicating how they were calculated

Our web collection on statistics for biologists contains articles on many of the points above.

Software and code

Policy information about availability of computer code

Data collection	NCBI GenBank database (https://www.ncbi.nlm.nih.gov/genbank/) was used to collect sequences for assay design.
Data analysis	Sequences were aligned using the MUSCLE algorithm in Geneious 2023.1.2 software, and oligos were designed using the software Primer Explorer version 5.0 (http://primerexplorer.jp/lampv5e/index.html) in combination with manual optimisation in Geneious. TTP and Ct values of LAMP and PCR reactions were automatically obtained from the software of the qPCR instruments used. Probit analysis was used to estimate probablity of obtaining a positive result with the Dragonfly platform.

For manuscripts utilizing custom algorithms or software that are central to the research but not yet described in published literature, software must be made available to editors and reviewers. We strongly encourage code deposition in a community repository (e.g. GitHub). See the Nature Portfolio guidelines for submitting code & software for further information.

Data

Policy information about availability of data

All manuscripts must include a data availability statement. This statement should provide the following information, where applicable:

- Accession codes, unique identifiers, or web links for publicly available datasets
- A description of any restrictions on data availability
- For clinical datasets or third party data, please ensure that the statement adheres to our policy

Data used in this study is available in the main manuscript and supplementary material.

Research involving human participants, their data, or biological material

Policy information about studies with human participants or human data. See also policy information about sex, gender (identity/presentation), and sexual orientation and race, ethnicity and racism.

Reporting on sex and gender	Sex and gender are described in the main manuscript (e.g.,Table 1)
Reporting on race, ethnicity, or other socially relevant groupings	No other relevant data have been included in the manuscript, and all samples were anonymised.
Population characteristics	Age groups are described in the main manuscript (e.g.,Table 1)
Recruitment	Approved by The Upper River Region in The Gambia was approved by the LSHTM Ethics Commitee (ref: 29611-1) and The Gambia Government/MRC Joint Ethics Committee (ref: 29611).
Ethics oversight	Collection of samples from asymptomatic individuals in The Upper River Region in The Gambia was approved by the LSHTM Ethics Commitee (ref: 29611-1) and The Gambia Government/MRC Joint Ethics Committee (ref: 29611). In Burkina Faso, sample collection from symptomatic patients in the Central West Region received the approval from The Comite d'Ethique pour la Recherche en Sante-Burkina Faso (ref: DELIBERATION N°2021-04-084) and the Comité d'Éthique Institutionnel pour la Recherche en Sciences de la Santé of IRSS (N/Réf. A07-2021/CEIRES); the approval for collecting samples from asymptomatic participants was granted by The Comite d'Ethique pour la Recherche en Sante-Burkina Faso (ref: DELIBERATION N° 2024-0361). Written informed consent was obtained from all research participants and/or guardians before recruitment.

Note that full information on the approval of the study protocol must also be provided in the manuscript.

Field-specific reporting

Please select the one below that is the best fit for your research. If you are not sure, read the appropriate sections before making your selection.

- [x] Life sciences
- [ ] Behavioural & social sciences
- [ ] Ecological, evolutionary & environmental sciences

For a reference copy of the document with all sections, see nature.com/documents/nr-reporting-summary-flat.pdf

Life sciences study design

All studies must disclose on these points even when the disclosure is negative.

Sample size	Sample size has been calculated based on the equation reported by Banoo et al. Considering a marginal error of 10% with a CI of 95%, and an estimated 90% sensitivity and specificity, the size of the study population is 672, including 146 positives by DBS-qPCR.
Data exclusions	No data was excluded.
Replication	For analytical testing, serial dilutions were tested at least in triplicates. Clinical samples were only tested once with Dragonfly given the available volume.
Randomization	Samples were randomly collected at the community level, including a total of 672 capillary blood specimens.
Blinding	Investigators were blinded to the nature of the samples (negative or positive to the different tested pathogens) during testing.

Reporting for specific materials, systems and methods

We require information from authors about some types of materials, experimental systems and methods used in many studies. Here, indicate whether each material, system or method listed is relevant to your study. If you are not sure if a list item applies to your research, read the appropriate section before selecting a response.

Materials & experimental systems

n/a	Involved in the study	
x		Antibodies
x		Eukaryotic cell lines
x		Palaeontology and archaeology
x		Animals and other organisms
x		Clinical data
x		Dual use research of concern
x		Plants

Methods

n/a	Involved in the study	
x		ChIP-seq
x		Flow cytometry
x		MRI-based neuroimaging

Plants

Seed stocks	N/A
Novel plant genotypes	N/A
Authentication	N/A
